# Factors Associated with Newly Developed Postoperative Neurological Complications in Patients with Emergency Surgery for Acute Type A Aortic Dissection

**DOI:** 10.3390/medicina60010027

**Published:** 2023-12-23

**Authors:** Mircea Robu, Irina Maria Margarint, Cornel Robu, Andreea Hanganu, Bogdan Radulescu, Ovidiu Stiru, Andrei Iosifescu, Silvia Preda, Mihai Cacoveanu, Cristian Voica, Vlad Anton Iliescu, Horațiu Moldovan

**Affiliations:** 1 Faculty of Medicine, Carol Davila University of Medicine and Pharmacy, 050474 Bucharest, Romania; cornel.robu@drd.umfcd.ro (C.R.); andreea.florea@drd.umfcd.ro (A.H.); bogdan.radulescu@umfcd.ro (B.R.); ovidiu.stiru@umfcd.ro (O.S.); iosifescuag@gmail.com (A.I.); silvia-mihaela.pieleanu@drd.umfcd.ro (S.P.); catalin.cacoveanu@gmail.com (M.C.); cristian.voica@drd.umfcd.ro (C.V.); vladanton.iliescu@gmail.com (V.A.I.); horatiu.moldovan@umfcd.ro (H.M.); 2 Prof. Dr. C.C. Iliescu Emergency Institute for Cardiovascular Diseases, 022322 Bucharest, Romania; 3 Department of Cardiac Surgery, Emergency Clinical Hospital for Children “Maria Skłodowska Curie”, 077120 Bucharest, Romania; 4 Neurology Department, Fundeni Clinical Institute, 022322 Bucharest, Romania; 5 Department of Cardiovascular Surgery, Emergency Clinical Hospital Bucharest, 014461 Bucharest, Romania; 6 Academy of Romanian Scientists, 050045 Bucharest, Romania

**Keywords:** dissection, thoracic aorta, ischemic stroke, hypoxia-ischemia, brain, spinal cord ischemia, delirium

## Abstract

*Background and Objectives*: Postoperative neurological complications (NCs) are an important cause of mortality in patients with acute type A aortic dissection (ATAAD). The aim of the study was to determine the association between intraoperative risk factors and newly developed postoperative NCs in patients after emergency surgery for ATAAD. *Materials and Methods*: A total of 203 patients requiring emergency surgery were included in the study. Patients with preoperative neurological dysfunction, deceased on the operating table or within the first 48 h after intensive care admission, with uncertain postoperative neurologic status or with incomplete records were excluded. *Results*: Mean age was 57.61 ± 12.27 years. Hyperlipidemia was the most frequent comorbidity (69%). A bicuspid aortic valve was present in 12.8% of cases, severe acute aortic regurgitation was present in 29.1% of patients, and cardiac tamponade was present in 27.1% of cases. The innominate artery was the most frequently dissected supra-aortic artery in 27.1% of cases. In 65% of cases, the primary entry tear was at the level of the ascending aorta. The incidence of newly developed postoperative NCs was 39.4%. The most common surgical technique performed was supra-coronary ascending aorta and hemiarch replacement, in 53.2% of patients. Using logistic regression, cardiopulmonary bypass time (OR = 1.01; 95% CI = 1.01–1.02; *p* < 0.001), aortic cross-clamp time over 3 h (OR = 2.71, 95% CI = 1.43–5.14, *p* = 0.002) and cerebral perfusion time (OR = 1.02; 95% CI = 1.002–1.03; *p* = 0.027) were independently associated with newly developed postoperative NCs. *Conclusions*: Based on the results of the study, all efforts should be made to reduce operative times. Using a simple surgical technique, like the supra-coronary ascending aorta and hemiarch technique, whenever possible, and using a simpler technique for cerebral perfusion like unilateral cerebral perfusion via the right axillary artery, could reduce operating times.

## 1. Introduction

Acute type A aortic dissection (ATAAD) is a lethal cardiac disease involving the aorta, with catastrophic effects on end-organ perfusion. The overall mortality rate is 5.8% at 48 h, with a decrease to 4.4% at 48 h in patients with emergency surgery [1,2].

Despite advances in surgical techniques (central aortic therapy) and the introduction of brain protection methods like cerebral perfusion and hypothermic circulatory arrest, the incidence of neurological complications (NCs) is high and ranges between 17 and 48% [3,4,5]. NCs after the emergency repair of an ATAAD are associated with higher in-hospital mortality, longer intensive care and hospital stays, and reduced long-term survival [6,7].

Ischemic stroke is the most clinically important NC, and we reported a 24.8% postoperative incidence in a previous study [8]. Spinal cord ischemia (SCI), hypoxic-ischemic encephalopathy (HE), and postoperative delirium (PD) are severe complications, but there are scarce data regarding their postoperative incidence after emergency surgery for ATAAD. In a retrospective study on 278 patients with type A and B aortic dissection, there was a 3.2% preoperative incidence of HE [9]. A systematic meta-synthesis literature review reported 67 cases of preoperative SCI caused by aortic dissection (type A and type B), while PD was present in 12–37% of patients with ATAAD [10,11].

Data regarding pre/postoperative NCs are heterogeneous, and there is wide variability in the categorization of NCs [11]. For example, in the reports by the International Registry of Aortic Dissection (IRAD), 17.1% of patients presented with “any focal neurological deficits” and 15% with “coma/altered consciousness” at admission. They reported that 29 and 8.5% of those patients presented these NCs during hospitalization, without information about surgical procedures [12]. The German Registry for Acute Aortic Dissection Type A (GERAADA) investigated hemiparesis/plegia, paraparesis/plegia, aphasia, and unconsciousness/coma as NCs [2]. Stroke is the only NC evaluated in the reports by The Nordic Consortium for Acute Type A Aortic Dissection (NORCAAD) and The Society of Thoracic Surgeons (STS) [6,13].

While preoperative risk factors for NCs in ATAAD can be considered endogenous, are patient related, and cannot be changed, intraoperative risk factors depend in great measure on the surgical team and strategy.

Despite the relative association between NCs and ATTAD, risk factors for newly developed postoperative NCs, especially intraoperative factors, remain uncertain (Table 1). For example, operative time was associated with new onset of NCs in the multi-center study GERAADA [13]. The Nordic Consortium for Acute Type A Aortic Dissection (NORCAAD) reports only total arch replacement as a risk factor for NCs [13], while The Society of Thoracic Surgeons (STS) reports that femoral cannulation increases the risk of stroke [6].

The aim of this study was to determine the association between different intraoperative parameters and newly developed postoperative NCs in patients after emergency surgery for ATAAD.

## 2. Materials and Methods

Between January 2017 and May 2023, 240 patients were transferred to our center for ATTAD management. The diagnosis of ATAAD was based on chest computer tomography (CT) with intravenous contrast. After a cardiology consultation in the emergency department with mandatory transthoracic echocardiography, patients who were candidates for emergency surgery were transferred into the operating room. Clinical characteristics and demographic data were collected from medical records and the electronic health system.

Inclusion criteria: patients with acute type A aortic dissection according the to Stanford classification were considered for emergency surgery.

Exclusion criteria: (1) patients with ischemic or hemorrhagic stroke documented on computed tomography (CT) scans prior to surgery; (2) clinical signs of stroke, paraplegia, delirium or any neurological dysfunction prior to surgery; (3) patients with history of transient ischemic stroke, ischemic or hemorrhagic stroke or any other neurological dysfunction; (4) patients who died in the operating room or within the first 48 h after intensive care admission; (5) patients in the postoperative setting for whom the neurological status could not be evaluated; (6) patients with incomplete medical records.

Immediate newly developed NCs were evaluated in the first 72 h of ICU stay.

Ischemic stroke was confirmed by head CT when clinical suspicion was raised in the postoperative period and after initial neurological examination. The Modified Rankin Scale (mRS) was used to quantify the degree of disability at discharge based on clinical charts and neurological examination.

Anterior spinal cord ischemia was diagnosed after a physical examination for paraplegia in the postoperative period. Spine MRI was used for confirmation, with T2-weighted image hyperintensity in the anterior horns being the hallmark finding.

Hypoxic-ischemic encephalopathy was diagnosed on head CT after neurological examination. CT findings were bilateral basal ganglia hypodensities, diffuse mass effect with effacement of the cerebral sulci, loss of the normal grey-white matter differentiation due to edema, and bilaterally decreased density in a watershed distribution.

Postoperative delirium was diagnosed by the Confusion Assessment Model for the Intensive Care Unit (CAM-ICU) criteria after all other causes for neurological dysfunction were excluded by the neurologist.

### 2.1. Surgical Technique

**Figure 1:**
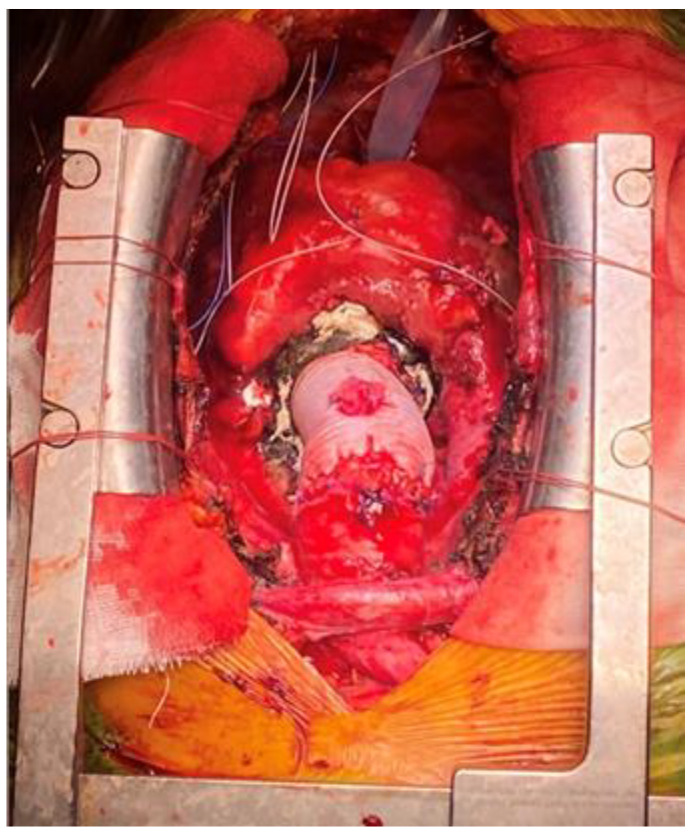
Supra-coronary ascending aorta replacement with a Dacron graft.

**Figure 2:**
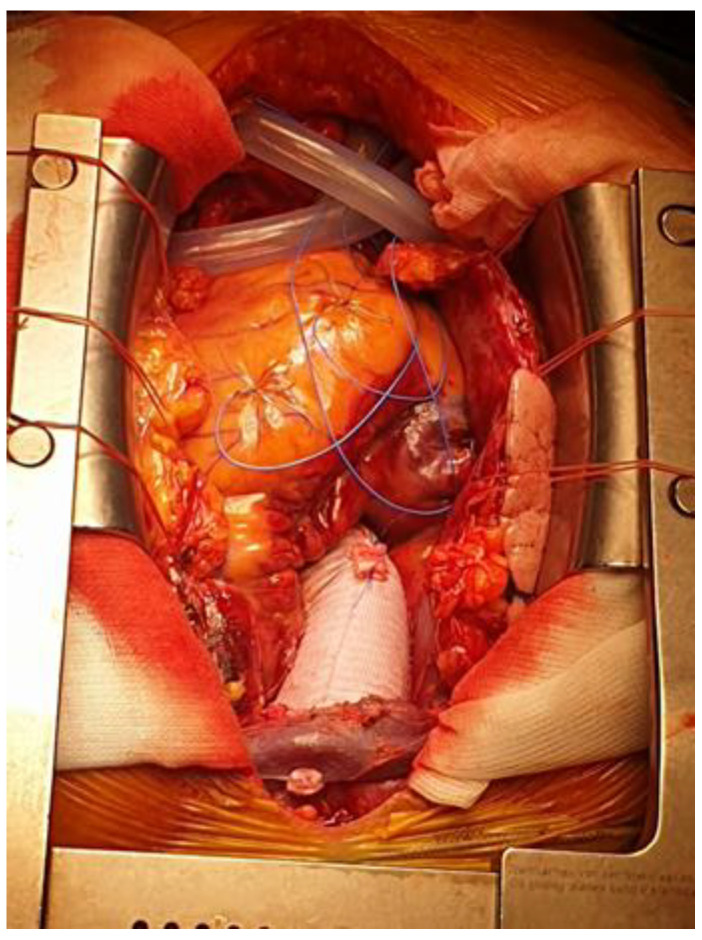
Ascending aorta and hemiarch replacement with a Dacron graft.

**Figure 3:**
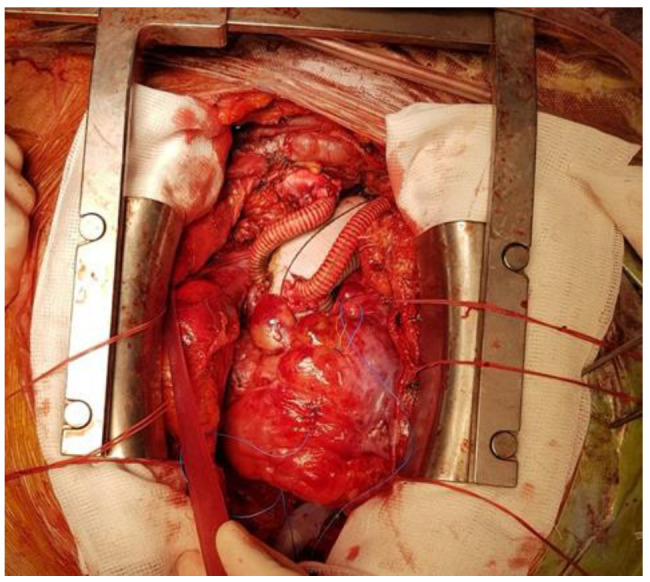
Total aortic arch replacement with a Dacron graft and separated reconnection of supra-aortic vessels.

Median sternotomy was the standard approach in all ATAAD cases after the induction of general anesthesia. The exposure of the axillary or femoral artery for arterial cannulation and a two-stage venous cannula in the right atrium would be considered for the aortic procedure only. Should an additional procedure such as mitral repair or replacement be required, then bicaval cannulation would be the obvious choice. Should any of the alternative arterial sites be considered unsuitable, then the direct cannulation of the dissected ascending aorta would be considered using a Seldinger technique under transesophageal echocardiographic guidance to identify the true aortic lumen. Anterograde and retrograde cold (4 °C) crystalloid cardioplegia was used for myocardial protection. Moderate hypothermia (25–28 °C) was considered when circulatory arrest was required. An open-distal anastomosis technique was preferred, using selective anterograde cerebral perfusion through the direct cannulation of the innominate and left common carotid artery. Near-infrared spectroscopy was used routinely for cerebral oxygenation monitoring. When the primary entry tear could not be addressed with a hemiarch approach, a total aortic arch replacement would be considered. Figure 1, Figure 2 and Figure 3 show the three main techniques used. The bilateral selective anterograde cerebral perfusion protocol is as follows: when the target temperature was achieved and circulatory arrest was initiated, the arch was opened, and two balloon-tipped cannulas were inserted under direct vision into the innominate artery and left common carotid artery. We used 13F or 15F cannulas depending on the vessel’s diameter. Each cannula was connected to a different pump with a separate pressure monitoring line. For the left side, the perfusion pressure was 60–70 mmHg with a flow of 180–230 mL/min. The right-side perfusion pressure was 60–70 mmHg with a flow between 150 and 200 mL/min. Perfusion parameters were adapted within the mentioned intervals to reach a target value of 50–70% on NIRS. Before completing the distal anastomosis, the cannulas were extracted one at a time, and the innominate and left common carotid arteries were snared until the completion of the anastomosis. After open-distal anastomosis was performed, an arterial cannula was inserted directly into the Dacron prosthesis, CPB was restarted, and the supra-aortic vessels were unclamped after de-airing. During the rewarming of the patient, the repair of the ascending aorta and or aortic root was performed. After weaning from CPB, hemostasis was achieved, epicardial leads were placed, and sternal osteosynthesis was achieved with steel wires.

### 2.2. Statistical Analysis

Statistical analysis was conducted with Wizard 2 Statistical Software for Mac OS (Wizard–Statistics and Analysis^®^, Raipur, Chattisgarh, India).

Summary statistics are presented as absolute numbers and percentages for categorical values and as mean ± standard deviation for continuous values. Our primary outcome was calculating the incidence of newly developed neurological complications in patients after emergency surgery for ATAAD. In order to investigate the association between different intraoperative factors and the development of new NCs, multivariable analysis was performed using logistic regression and taking into account a model that included variables achieving a *p*-value < 0.1 in univariate analysis. A predictive modeling strategy with the backward stepwise method of entering data was then used. Variables included in the univariate analysis were the following: age, male sex, arterial hypertension, diabetes, hyperlipidemia, chorionic kidney disease, preoperative atrial fibrillation, cardiac tamponade at admission, severe aortic regurgitation, severe left ventricle dysfunction, presence of bicuspid aortic valve, a primary intimal tear in the ascending aorta, aortic arch, ascending aorta and aortic arch or no entry tear found in the ascending aorta or aortic arch (based on the preoperative chest CT), the dissection of one, two or three supra-aortic vessels, and reintervention for mediastinal bleeding. Intraoperative indexes were type of surgery (aortic arch/no aortic arch surgery, Wheat procedure, aortic root surgery, supra-coronary ascending aorta replacement, hemiarch replacement, total arch replacement, innominate artery implantation, innominate artery and left common carotid artery implantation, combined procedures), axillary or femoral artery cannulation, cardiopulmonary bypass time, aortic cross-clamp time, and cerebral perfusion time. The logistic regression results are presented as odds ratios (ORs) with confidence limits and *p*-values. *p* < 0.05 was considered statistically significant. An independent *t*-test or Chi-square test was used for comparing the neurologically complicated and uncomplicated groups.

## 3. Results

### 3.1. General Characteristics of Study Population

After applying exclusion criteria, a total of 203 patients with ATAAD and emergency surgery were included in the study. In total, a number of thirty-seven patients were excluded (five patients with massive ischemic stroke, three patients with hemorrhagic stroke, four patients with unresponsive cardiac arrest at admission, three patients with history of neurological dysfunction, five patients with irreversible peripheric ischemia, four patients with ischemic stroke on preoperative CT, thirteen patients died on the operating table, ten because of hemorrhagic stroke and three patients had myocardial infarction. The mean age was 57.61 ± 12.27 years, 67% of patients were males, and the mean Euroscore was 9.03 ± 2.63 (Table 2). The time from diagnosis of ATAAD to surgery was 4.89 ± 4.37 h. Hyperlipidemia was the most frequent comorbidity (69%), and preoperative atrial fibrillation was seen in 7.4% of cases. Thoracic pain was present in 175 patients (8.2%). A history of aortic aneurysm was identified in 22.16%, while the mean maximum aortic diameter was 6.19 ± 1.19 mm. Data from preoperative transthoracic echocardiography showed the presence of a bicuspid aortic valve in 12.8% of cases, severe acute aortic regurgitation in 29.1% of patients, and cardiac tamponade in 27.1% of cases. Based on preoperative CT, the innominate artery was the most frequently dissected supra-aortic artery in 27.1% of cases, while the dissection of all three supra-aortic vessels was encountered in 9.9% of patients. The postoperative death rate was 16.74% (34 patients). Causes of death based on autopsy reports were the following: septic shock in 12 patients (25.5%), cardiogenic shock in 11 patients (32.4%), mixed shocked in 9 cases (26.4%) and hemorrhagic shock in 2 patients (5.9%).

### 3.2. Intraoperative Data

The intraoperative data (Table 3) show a mean cardiopulmonary bypass time of 206.28 ± 63.22 min and an aortic cross-clamp time of 116.1 ± 38.4 min, and 76.4% of cases required cerebral perfusion, with a mean cerebral perfusion time of 29.62 ± 22.27 min. The most frequent procedure in the group with no intervention at the level of the aortic arch was supra-coronary ascending aorta replacement with a Dacron graft in 18.7% of cases. In the group with arch intervention, the most common type of surgery was supra-coronary ascending aorta and hemiarch replacement, with moderate hypothermic circulatory arrest and cerebral perfusion in 53.2% of cases. Total arch replacement was performed in 4.4% of cases. Additional procedures were required in 31 patients: coronary artery bypass grafting in 5.4% of cases, mitral valve replacement in 2.46% of cases, mitral valve repair in 1.47% of cases, peripheral V-A ECMO in two patients, non-coronary sinus reconstruction in two patients, a femoro-femoral bypass in one patient, and aortic coarctation repair in one patient. Axillary artery cannulation was performed in 64.5% of cases, followed by femoral artery cannulation in 31.5% of patients.

### 3.3. Characteristics of Patients with Neurological Complications

The incidence of newly developed postoperative NCs was 39.4% (80 patients). Ischemic stroke had an incidence of 23.6% (48 patients). Postoperative delirium was encountered in 21 patients, with an incidence of 10.3%, and there were 9 cases (4.4%) of hypoxic encephalopathy. Two (1%) patients developed spinal cord ischemia in the postoperative period.

No difference was observed in age, frequency of preoperative factors, and surgical technique between neurologically complicated and uncomplicated groups (Table 4). Patients with newly developed postoperative NCs had a longer cardiopulmonary bypass time (*p* = 0.004) and cerebral perfusion time (*p* < 0.001) and had a higher frequency of the dissection of two supra-aortic vessels (*p* = 0.048) and primary entry tears in the aortic arch (*p* = 0.014). The incidence of death in the NC group was 23.8% (septic shock in eight cases, mixed shock in six cases and cardiogenic shock in five cases).

#### 3.3.1. Ischemic Stroke

Patients with ischemic stroke (Table 4) were older than patients without ischemic stroke (*p* = 0.011), had a higher frequency of the dissection of two supra-aortic vessels (*p* = 0.03) and entry tears in the ascending aorta and arch (*p* = 0.001), and had a longer cardiopulmonary bypass time (*p* = 0.008) and cerebral perfusion time (*p* = 0.001). A significant difference was found in the frequency of death in the ischemic stroke group (*p* = 0.001). Postoperative ischemic stroke was diagnosticated in 23.64% of patients (Table 5). The incidence of death was 33.34% in this group. Anterior circulation was involved in 47.91% of cases, posterior circulation was involved in 20.83% of cases, and both anterior and posterior circulation were affected in 16.67% of patients.

The right carotid artery was the most affected vessel of the anterior circulation, summing 25% of the cases. In the posterior circulation, the most affected vessel was the right vertebrobasilar artery, in 8.33% of cases. Supra-aortic vessel dissection was observed in 39.6% of patients with ischemic stroke. The most frequently dissected branch was the innominate artery, in 35.4% of cases. Six patients (33.34%) died in the ischemic stroke group, and eleven (22.91%) had no disability or a slight disability at discharge (modified Rankin scale between 0–2).

#### 3.3.2. Hypoxic Encephalopathy

Hypoxic encephalopathy had an incidence of 4.4% (nine patients). The mean age was 59.22 ± 13.53 years. Systolic hypotension related to cardiac tamponade was observed in four patients and related to acute severe aortic regurgitation in two patients. Two patients exhibited the involvement of the supra-aortic vessels, both of them with dissection of the innominate artery and the left common carotid artery, without the dissection of the subclavian arteries. Three patients had a primary entry tear localized at the level of the aortic arch, and five patients had aortic arch surgery. The mean cardiopulmonary bypass time was 239.11 ± 49.55 min, the aortic cross-clamp time was 126.11 ± 48.53 min, and the cerebral perfusion time was 24.67 ± 22.37 min. One patient did not regain consciousness and died of septic shock; the remaining patients were successfully transferred to physical rehabilitation centers after intensive care management was no longer required.

#### 3.3.3. Postoperative Delirium

Postoperative delirium was observed in 21 patients (10.3%). The mean age was 58.46 ± 12.39 years; 66.7% had a history of arterial hypertension and 9.5% had diabetes mellitus. Cardiac tamponade at admission was present in 19% of cases, and severe acute aortic regurgitation was seen in 42.9% of cases. In 47.6% of cases, aortic arch surgery was performed, and 9.5% of patients had total arch replacement. Supra-aortic vessel involvement was seen in 23.8% of patients. The incidence of innominate artery dissection was 23.8%, and 14.3% had both innominate artery and left common carotid artery involvement. The mean cardiopulmonary bypass time was 216.29 ± 51.81 min, aortic cross-clamp time was 117.38 ± 29.77 min, and cerebral perfusion time was 28.81 ± 14.8 min. There was a significant difference in the incidence of aortic root surgery (*p* = 0.025), and patients with postoperative delirium had a higher frequency of cardiopulmonary bypass time over 3 h (*p* = 0.049). One patient died group from mixed shock (cardiogenic and septic shock).

#### 3.3.4. Spinal Cord Ischemia

Spinal cord ischemia was diagnosed in two patients (2.5%). Both patients were male and had ascending aorta and hemiarch replacement with entry tears in the ascending aorta. Cardiopulmonary bypass time was 168 ± 50.2 min, aortic cross-clamp time was 88 ± 21.21 min, and cerebral perfusion time was 33 ± 15.56 min. One patient had innominate artery dissection and the other patient had both innominate artery and left common carotid artery involvement. Both patients survived, one patient with a partial recovery from paraplegia at discharge and the other patient with no recovery.

### 3.4. Statistical Analysis (Logistic Regression)

The results of the univariate analysis of variables with a *p*-value < 0.1 and multifactor logistic regression analysis results are presented in Table 6. After backward selection only age (OR = 1.03; 95% CI = 1.01–1.06; *p* = 0.013) and hyperlipidemia (OR = 2.08; 95% CI = 1.05–4.12; *p* = 0.035) were included in the final model. Cardiopulmonary bypass time was associated with newly developed postoperative NCs in univariate analysis (OR = 1.01; 95% CI = 1.003–1.02; *p* = 0.001) and after model adjustment was an independent risk factor for postoperative NCs (OR = 1.01; 95% CI = 1.01–1.02; *p* < 0.001). Aortic cross-clamp time was not associated with newly developed postoperative NCs in univariate analysis (*p* = 0.06); however, aortic cross-clamp time over 3 h was associated with newly developed postoperative NCs in univariate analysis (OR = 2.2; 95% CI = 1.2–4.04; *p* = 0.011) and after model adjustment was an independent risk factor for postoperative NCs (OR = 2.71; 95% CI = 1.43–5.14; *p* = 0.002). Cerebral perfusion time was associated with newly developed postoperative NCs in univariate analysis (OR = 1.01; 95% CI = 1.001–1.03; *p* = 0.042), and after adjustment for age and hyperlipidemia, was an independent risk factor for newly developed postoperative NCs (OR = 1.02; 95% CI = 1.002–1.03; *p* = 0.027).

## 4. Discussion

Acute type A aortic dissection (ATAAD) represents a catastrophic event with high mortality (5.8% at 48 h) and morbidity in the absence of emergency surgery [1,2,9].

Despite advances in surgical techniques and brain protection methods, neurological complications in this group of patients rage from 17 to 48% [3,4,5]. In our study, the incidence of newly developed postoperative NCs was 39.4%. The high variability in the reported incidence of NCs can be due to difficulties in recording them in critically ill patients [4]. Data regarding pre- and postoperative NCs are heterogeneous and there is wide variability in the categorization of NCs [2,13,14].

Ischemic stroke is the most clinically important newly developed perioperative NC, with a hospital mortality rate six times greater than patients without stroke [14,15,16]. The incidence of ischemic stroke in this study was 23.6%, being the most frequent NC (60%), in accordance with previous reports [3,4,5]. Ischemic stroke tends to be more frequent in the anterior circulation (47.91%) than the posterior one (20.83%) and is predominantly right-sided in both cases (25% right carotid artery anterior, 8.33% right vertebrobasilar artery). Supra-aortic vessel involvement was observed in 39.6% of patients with ischemic stroke, and the frequency of two-vessel involvement was significantly higher in the NC group (16.7% vs. 6.5%; *p* = 0.03). One reason for the right-side dominance of lesions can be that hydraulic stress is greatest in the right lateral wall of the ascending aorta [17]. However, 60.4% of patients with ischemic stroke did not have supra-aortic vessel involvement, suggesting there are other mechanisms present in the pathogenesis of ischemic stroke. Thromboembolism, microembolism, or hypotension may be such factors. Patients with ischemic stroke were significantly older (61.12 ± 9.27 vs. 56.52 ± 12.9; *p* = 0.011), which could be explained by the aggravation of atherosclerosis with the increase in age [18]. Sixteen patients (33.34%) with ischemic stroke died in the intensive care unit, and 43.75% had a mRs between 3 and 5, confirming the high mortality and morbidity of this postoperative complication.

Hypoxic-ischemic encephalopathy is a severe complication of global ischemia, with a spectrum of disability that ranges from complete recovery from coma to death, while data on long term outcome are rare [19,20,21]. The incidence of hypoxic-ischemic encephalopathy in ATAAD is also rare. A retrospective study on 278 patients with type A and B aortic dissection found a preoperative incidence of HE of 3.2% [9]. Blanco et al. reported 5 patients with preoperative hypoxic-ischemic encephalopathy in 24 patients with ATAAD [4]. In our study, the incidence was 4.4% (nine patients). Systolic hypotension related to cardiac tamponade was observed in four patients (44.4%) and related to acute severe aortic regurgitation in two patients. Two patients exhibited the involvement of the supra-aortic vessels, both of them with dissection of the innominate artery and left common carotid artery, without the involvement of the subclavian arteries. To date, surgery is the best approach to protect against definitive neurological deficits when cerebral malperfusion is suspected [22]. Axillary cannulation and bilateral anterograde cerebral perfusion should always be considered in these cases [22]. In our study, six out of nine patients with hypoxic-ischemic encephalopathy had axillary artery cannulation and bilateral anterograde cerebral perfusion. One patient did not regain consciousness and died of septic shock.

Spinal cord ischemia in ATAAD is also a rare condition, and little data are available on its incidence [23]. Spinal cord ischemia presents as anterior spinal cord syndrome in 87.2% of cases, while SCI in the territory of the posterior spinal artery is very rare [24]. There are a few data regarding SCI in ATTAD. One study reports that 1% of patients presenting with ATAAD have SCI [25] A systematic meta-synthesis literature review found 67 cases of SCI caused by aortic dissection (type A and type B). Out of 19 patients having undergone emergency surgery, full recovery in 13 patients, residual paraplegia in 4 patients, and death in 2 patients was observed. The overall mortality was 57.14% in patients presenting with SCI in the setting of ATAAD [23]. Two patients developed SCI in our study and both of them were discharged, one without recovery and the other with a partial recovery from paraplegia.

Postoperative delirium is a severe brain disorder characterized by inattention, disturbed thinking and altered levels of consciousness [25,26]. A meta-analysis of 12 studies from 2016 to 2022 found that low oxygen levels, prolonged mechanical ventilation, renal dysfunction, low hemoglobin levels and prolonged intensive care stay are risk factors for PD in ATAAD [21]. Incidence in cardiac surgery is high (32.5 and 52%) and involves between 12 and 37% of patients with ATAAD [10,11]. In our study, 21 (10.3%) patients developed this NC. Only one patient died in our study (mixed cardiogenic and septic shock).

Despite the relative association between NCs and ATTAD, risk factors for newly developed postoperative NCs, especially intraoperative factors, remain uncertain. A multi-center study (GERAADA) with 2137 patients found that operative time was associated with new onset of NCs [13]. Another study enrolled 501 patients with aortic arch surgery, moderate hypothermic circulatory arrest, and selective anterograde cerebral perfusion. They found that permanent NCs were associated with operation time and temporary NCs were associated with the duration of circulatory arrest [27]. The Nordic Consortium for Acute Type A Aortic Dissection reports only total arch replacement as a risk factor for NCs [13], while the Society of Thoracic Surgeons reports that femoral cannulation increases the risk of stroke [6].

While preoperative risk factors for NCs in ATAAD can be considered endogenous, are patient-related, and cannot be changed, intraoperative risk factors depend in great measure on the surgical team and strategy.

Preoperative risk factors associated in univariate analysis with newly developed postoperative NCs in this study were age (OR = 1.02; 95% CI = 0.1–1.05; *p* = 0.025), aortic arch tear (OR = 2.36, 95% CI = 1.29–4.35; *p* = 0.015) and ascending aorta and aortic arch entry tear (OR = 5.25; 95% CI = 1.77–15.61; *p* = 0.009). Older patients have a well-documented risk of NCs, probably due to advanced atherosclerotic lesions and a higher susceptibility to brain hypoperfusion once the aortic dissection occurs [18,28]. Regarding primary entry tears in the aortic arch, both in the aortic arch and ascending aorta, neither GERAADA, NORCAD nor IRCAD found any association with NCs in ATAAD [2,12,13]. It is possible that normal flow in the supra-aortic vessels could be more frequently reduced by an entry tear in the aortic arch causing dynamic or static obstruction by the intimal flap [29].

Cardiopulmonary bypass time (OR = 1.011; 95% CI = 1.01–1.02; *p <* 0.001), aortic cross-clamp time over three hours (OR = 2.71; 95% CI = 1.43–5.14; *p* = 0.002) and cerebral perfusion times (OR = 1.02; 95% CI = 1.002–1.03; *p* = 0.027) were independent risk factors associated with newly developed postoperative NCs in the present study. This is in accordance with the GERAADA study, where increased operating times were associated with NCs. They found that cardiopulmonary bypass time and circulatory arrest time were independent risk factors [2]. Aortic cross-clamp time was not investigated, and neuroprotective strategies such as anterograde cerebral perfusion were not associated with de novo NCs [2]. We reported in a previous study that bilateral selective anterograde cerebral perfusion over 40 min is a risk factor for new postoperative ischemic stroke [8]. Permanent neurological dysfunction was associated with longer operation time, while increasing hypothermic circulatory arrest was associated with temporary neurologic dysfunction, in a study of 501 patients with aortic arch surgery [27].

There were no differences between the neurologically complicated and uncomplicated groups regarding the type of surgery or site of cannulation. The most frequent procedure was ascending aorta and hemiarch replacement with moderate hypothermic circulatory arrest and bilateral anterograde selective cerebral perfusion, and the most frequent cannulation site was the axillary artery. This result is supported by data from the current literature [2,30,31] and suggests that cerebral flow can be influenced by multiple factors such as flow direction, pressure, temperature, or atherosclerosis lesions.

## 5. Conclusions

We analyzed data from 203 patients who had emergency surgery for ATAAD without neurological dysfunction at presentation in order to identify intraoperative risk factors for new postoperative NCs. Newly developed postoperative NCs in our study were ischemic stroke, delirium, spinal cord ischemia and hypoxic-ischemic encephalopathy. Cardiopulmonary bypass time, aortic cross-clamp time over 3 h, and cerebral perfusion time were independently associated with newly developed postoperative NCs using multivariable analysis. The type of surgery and cannulation site were not associated with newly developed NCs in univariate analysis. These results emphasize that perioperative management, the technical facilities of each center, and surgical strategies are important in the prognosis of these patients. All efforts should be made to minimize operative times, considering that the study results prove that longer operative times are associated with an increased risk of NCs.

Considering the results of this study regarding reducing operating times, some recommendations could be: using a simple surgical technique like the supra-coronary ascending aorta and hemiarch technique whenever possible; performing distal anastomosis with the aortic clamp in place if no entry tear is present in the aortic arch; using direct ascending aorta/aortic arch cannulation using the Seldinger technique for the rapid institution of CBP and optimal flow during CPB; and using a simpler technique for cerebral perfusion like unilateral cerebral perfusion via the right axillary artery. Also, we consider that a consensus is required to define neurological dysfunction in ATAAD patients and could be essential for the easier identification of risk factors.

## Figures and Tables

**Table 1:** Risk factors associated with NCs.

	Risk Factor	OR	95% CI	*p*
GERAADA [2]	Three or more malperfused organs	2.206	1.278–3.810	0.038
	Dissection of supra-aortic vessels	1.468	1.095–1.969	0.0103
	Operative time	1.002	1.001–1.003	0.0001
	Cardiopulmonary bypass time	1.002	1.001–1.004	0.0005
	Circulatory arrest time	1.009	1.003–1.015	0.0017
NORCAAD [13]	Cerebral malperfusion	4.28	2.56–7.17	<0.001
	Cardiopulmonary bypass time	1.19	1.11–1.26	<0.001
	Pericardial tamponade	1.85	1.12–3.05	0.015
	Cardiogenic shock	2.45	1.20–4.98	0.013
STS [6]	Hypertension	1.35	1.10–1.64	0.003
	Syncope	1.56	1.24–1.96	<0.001
	Cardiopulmonary bypass time	1.001	1.001–1.002	0.003
	Femoral artery cannulation, no cerebral perfusion, no arch repair			<0.001

**Table 2:** General characteristics of patients with emergency surgery for ATAAD; mean ± SD; *n* (%).

Parameter (Unit)	*n* = 203 (100%)
Age (years)	57.61 ± 12.27
Gender (male)	136 (67%)
Euroscore	9.03 ± 2.63
Time from diagnostic to surgery (hours)	4.89 ± 4.37
Hypertension	130 (64%)
Diabetes mellitus	13 (6.4%)
Hyperlipidemia	140 (69%)
Chronic kidney disease	14 (6.9%)
Hemodynamically stable patients	149 (73.4%)
Preoperative atrial fibrillation	15 (7.4%)
Cardiogenic shock	22 (9.56%)
ST elevation myocardial infarction	7 (3.44%)
Mesenteric ischemia	5 (2.46%)
Lower leg ischemia	32 (15.76%)
Root aneurysm	11 (5.41%)
Thoracic pain	175 (86.2%)
Abdominal pain	32 (15.76%)
History of aortic aneurysm	45 (22.16%)
Acute heart failure	31 (15.27%)
Bicuspid aortic valve	26 (12.8%)
Maximum diameter	6.19 ± 1.19
Severe acute aortic regurgitation	59 (29.1%)
Severe left ventricle dysfunction (LVEF < 30%)	4 (1.97%)
Moderate left ventricle dysfunction (LVEF 30–40%)	5 (2.5%)
Mild left ventricular dysfunction (LVEF 40–50%)	111 (54.67%)
Cardiac tamponade at admission	49 (24.1%)
Dissection of innominate artery	55 (27.1%)
Dissection of innominate artery and left common carotid artery	32 (15.8%)
Dissection of all supra-aortic vessels	20 (9.9%)
Primary entry tear in the ascending aorta	132 (65%)
Ascending aorta and aortic arch	17 (8.4%)
Ascending arch	66 (32.5%)
No entry tear found	23 (11.3%)

LVEF: left ventricular ejection fraction.

**Table 3:** Intraoperative data; mean ± SD; *n* (%).

Operative Data	*n* = 203 (100%)
Type of surgery	
No-arch surgery	76 (37.4%)
Supra-coronary ascending aorta replacement	38 (18.7%)
Wheat procedure	6 (3%)
Aortic root and ascending aorta replacement	11 (5.4%)
Arch surgery	127 (62.6%)
Ascending aorta and hemiarch replacement	108 (53.2%)
Ascending aorta and total arch replacement	9 (4.4%)
Aortic root, ascending aorta and hemiarch replacement	21 (10.3%)
Implantation of innominate artery	6 (3%)
Implantation of innominate artery and left common carotid artery	8 (3.9%)
Combined procedure	31 (15.3%)
CABG	11 (5.4%)
Mitral valve prosthesis	5 (2.46%)
Mitral valve repair	3 (1.47%)
Peripheral V-A ECMO	2 (0.98%)
Non-coronary sinus reconstruction	2 (0.98%)
Femoro-femoral bypass	1 (0.5%)
Aortic coarctation repair	1 (0.5%)
Cannulation site	
Axillary artery	131 (64.5%)
Femoral artery	64 (31.5%)
Dissected ascending aorta	7 (3.4%)
Aortic arch	1 (0.5%)
Cardiopulmonary bypass time (min)	206.28 ± 63.22
Aortic cross-clamp time (min)	116.1 ± 38.4
Bilateral selective anterograde cerebral perfusion (min)	29.616 ± 22.27
Cerebral perfusion and deep hypothermic circulatory arrest	155 (76.4%)
Subsequent intervention for mediastinal bleeding	50 (24.6%)

CABG: coronary artery bypass grafting; V-A ECMO: veno-arterial extracorporeal membrane oxygenator.

**Table 4:** Comparison between the patients with type A aortic dissection with and without neurological complications: mean ± SD; *n* (%).

	NC (+)	NC (−)	*p*	Stroke (+)	Stroke (−)	*p*
	*n* = 80	*n* = 123		*n* = 48	*n* = 155	
*Preoperative*						
Age	60.02 ± 10.71	56.04 ± 12.99	0.098	61.12 ± 9.27	56.52 ± 12.9	0.011
Male	54 (67.5%)	82 (66.7%)	0.902	30 (62.5%)	106 (68.4%)	0.448
Hypertension	49 (61.3%)	81 (65.9%)	0.504	29 (60.4%)	101 (65.2%)	0.549
Diabetes mellitus	4 (5%)	9 (7.3%)	0.51	2 (4.2%)	11 (7.1%)	0.469
Hyperlipidemia	61 (76.2%)	79 (64.2%)	0.07	38 (79.2%)	102 (65.2%)	0.08
Chronic kidney disease	6 (7.5%)	8 (6.5%)	0.784	3 (6.2%)	11 (7.1%)	0.84
Cardiac tamponade	22 (27.5%)	27 (22%)	0.367	13 (27.1%)	36 (23.2%)	0.585
Severe aortic regurgitation	22 (27.5%)	37 (30.1%)	0.692	11 (22.9%)	48 (31%)	0.283
Preoperative atrial fibrillation	8 (10%)	7 (5.7%)	0.251	5 (10.4%)	10 (6.5%)	0.359
Dissection of supra-aortic vessels	26 (32.5%)	39 (31.7%)	0.906	19 (39.6%)	46 (29.7%)	0.199
One vessel	10 (12.5%)	22 (17.9%)	0.303	6 (12.5%)	26 (16.6%)	0.478
Two vessels	11 (13.8%)	7 (5.7%)	0.048	8 (16.7%)	10 (6.5%)	0.03
Three vessels	9 (11.2%)	11 (9%)	0.603	8 (16.7%)	12 (7.8%)	0.072
Bicuspid aortic valve	9 (11.2%)	17 (13.8%)	0.592	5 (10.4%)	21 (13.5%)	0.57
LVEF < 30%	3 (3.8%)	2 (1.6%)	0.34	2 (4.2%)	3 (1.9%)	0.383
LVEF 30–40%	2 (2.5%)	4 (3.3%)	0.757	2 (4.2%)	4 (2.6%)	0.571
LVEF 40–50%	32 (40%)	37 (30.1%)	0.145	20 (41.7%)	49 (31.6%)	0.199
*Intraoperative*						
No arch surgery	29 (36.2%)	47 (38.2%)	0.778	14 (29.2%)	62 (40%)	0.175
Wheat procedure	1 (1.2%)	5 (4.1%)	0.247	1 (2.1%)	5 (3.2%)	0.683
Aortic root surgery	15 (18.8%)	18 (14.6%)	0.437	6 (12.5%)	27 (17.4%)	0.42
Supra-coronary ascending aorta replacement	12 (15%)	26 (21.1%)	0.273	7 (14.6%)	31 (20%)	0.401
Arch surgery	51 (63.7%)	76 (61.8%)	0.778	34 (70.8%)	93 (60%)	0.175
IA implantation	3 (3.8%)	3 (2.4%)	0.59	1 (2.1%)	5 (3.3%)	0.683
IA and LCCA implantation	4 (5%)	4 (3.3%)	0.532	3 (6.3%)	5 (3.2%)	0.347
Total arch replacement	5 (6.2%)	4 (3.3%)	0.311	3 (6.3%)	6 (3.9%)	0.484
Hemiarch replacement	42 (52.5%)	66 (53.7%)	0.872	29 (60.4%)	79 (51%)	0.252
Combined procedures	9 (11.2%)	22 (17.9%)	0.199	4 (8.3%)	27 (17.4%)	0.126
Axillary artery cannulation	56 (70%)	49 (61%)	0.189	34 (70.8%)	97 (62.6%)	0.296
Femoral artery cannulation	24 (30%)	42 (34.1%)	0.583	14 (29.2%)	52 (33.5%)	0.571
Ascending aorta entry tear	48 (60%)	84 (68.3%)	0.226	32 (66.7%)	100 (74.5%)	0.785
Aortic arch entry tear	34 (42.5%)	32 (26%)	0.014	21 (43.8%)	45 (29%)	0.057
Ascending aorta and arch entry tear	12 (15.2%)	5 (4.1%)	0.005	10 (21.3%)	7 (4.5%)	<0.001
No entry tear	10 (12.5%)	13 (10.6%)	0.671	5 (10.4%)	18 (11.6%)	0.819
Cardiopulmonary bypass time	224.8 ± 66.62	194.24 ± 58.07	0.004	228.39 ± 74.93	199.43 ± 57.69	0.008
Aortic cross-clamp time	122.47 ± 41.71	111.96 ± 35.66	0.12	125.1 ± 45.53	113.32 ± 35.62	0.322
Cerebral perfusion time	33.6 ± 22.25	27.02 ± 21.98	<0.001	36.89 ± 24.27	27.36 ± 21.18	0.001
Cerebral perfusion	65 (81.2%)	90 (73.2%)	0.186	39 (81.2%)	116 (74.2%)	0.316
Death	19 (23.8%)	17 (13.8%)	0.07	16 (33.3%)	20 (12.9%)	0.001

NC: neurological complication; IA: innominate artery; LCCA: left common carotid artery; LVEF: left ventricular ejection fraction.

**Table 5:** Stroke characteristics, sites of arterial dissection and discharged mRS in patients with emergency surgery for ATAAD; *n* (%).

Stroke Characteristics	*n* = 48 (23.64%)
Anterior circulation	23 (47.91%)
Left carotid	9 (18.75%)
Right carotid	12 (25%)
Bilateral carotid	2 (4.16%)
Posterior circulation	10 (20.83%)
Right vertebrobasilar	4 (8.33%)
Left vertebrobasilar	3 (6.25%)
Bilateral	3 (6.25%)
Anterior and posterior circulation	15 (31.25%)
Supra-aortic vessels dissection	19 (39.6%)
Innominate artery	17 (35.4%)
Left common carotid artery	15 (31.2%)
Left subclavian artery	11(22.9%)
Discharge mRS	
0–2	11 (22.91%)
3–5	21 (43.75%)
6	16 (33.34%)

mRS: modified Rankin scale, ATAAD: acute type A aortic dissection.

**Table 6:** Factors associated with newly developed NCs (Multivariable analysis).

	Univariate Analysis			Multivariable Analysis		
	OR	95% CI	*p*	OR	95% CI	*p*
Age (years)	1.02	0.1–1.05	0.025			
Aortic arch tear	2.36	1.29–4.35	0.015			
Ascending aorta and aortic arch tear	5.25	1.77–15.61	0.009			
Dissection of two supra-aortic vessels	2.64	0.98–7.13	0.055			
Hyperlipidemia	1.79	0.95–3.37	0.072			
Cardiopulmonary bypass time	1.01	1.003–1.02	0.001	1.011	1.01–1.02	<0.001
Aortic cross-clamp time	1.007	1.003–1.015	0.06			
Aortic cross-clamp over 3 h	2.2	1.2–4.04	0.011	2.71	1.43–5.14	0.002
Cerebral perfusion time	1.01	1.001–1.03	0.04	1.02	1.002–1.03	0.027

## Data Availability

The data presented in this study are available on reasonable request from the corresponding author.
